# Verifying Pressure of Water on Dams, a Case Study

**DOI:** 10.3390/s8095376

**Published:** 2008-09-03

**Authors:** Temel Bayrak

**Affiliations:** Karadeniz Technical University, Engineering Faculty, Dept. of Geodesy and Photogrammetry, 61080, Trabzon, Turkey; E-Mail: tbayrak@ktu.edu.tr; Tel.: +90-462-377-2763; Fax: +90-462-328-0918

**Keywords:** Dams, Safety and Hazards, Statistical Analysis

## Abstract

Sensing and monitoring deformation pattern of dams is often one of the most effective ways to understand their safety status. The main objective of the present study is to find the extent to which rising reservoir level affects the mechanism of deformation of the Yamula dam under certain changes in the reservoir level conditions during the first filling period. A new dynamic deformation analysis technique was developed to analyze four geodetic monitoring records consisting of vertical and horizontal displacements of nine object points established on the dam and six reference points surrounding it, to see whether the rising reservoir level is responsible for the vertical and horizontal deformations during the first filling period. The largest displacements were determined in the middle points of the dam construction. There is an apparent linear relationship between the dam subsidence and the reservoir level. The dynamic deformation model was developed to model this situation. The model infers a causative relationship between the reservoir level and the dam deformations. The analysis of the results determines the degree of the correlation between the change in the reservoir level and the observed structural deformation of the dam.

## 1. Introduction

The failure of several major dams causing great destruction and high death tolls has led to a systematic monitoring of major dams and reservoirs in order to ensure their structural integrity, the prevention of major damage, and especially, the safety of the public. Driven by the development of measuring and analysis techniques, the goal of geodetic deformation analysis nowadays is to proceed from a merely phenomenological description of the deformation of an object to the analysis of the process which caused the deformation [1]. Analysis of deformations of any type of a deformable body includes geometrical analysis and physical interpretation. Geometrical analysis describes the change in shape and dimensions of the monitored object. The ultimate goal of the geometrical analysis is to determine in whole deformable object the displacements and strain fields in the space and domains. Physical interpretation is to establish the relationship between the causative factors (loads) and the deformations. This can be determined either by statistical method, which analyses the correlation between the observed deformations and loads [2].

In this study, as an example, the effect of pressure of water on the dam settlement during the first filling of reservoir is shown, with geodetic monitoring results using statistical methods, which analyse the correlation between the observed deformations and loads. Thus, we analyze four geodetic records covering the first filling period, describe the subsidence of the body of a large size earth fill dam, called The Yamula, and try to investigate the effect of the increase of the reservoir level on the dam. The problem is, thus, how the rising water level of the reservoir effects the vertical deformations of the dam during the first filling period. A developed deformation model was used to answer this question. The dynamic model contains the calculation of a parameter of the rising reservoir level, which shows the geometric signature of the physical effect. Finally, the acceleration effect of rising level in large reservoirs on the dam deformations was investigated.

## 2. The Yamula dam and geodetic deformation monitoring system

The Yamula Dam, on the Kızılırmak River, is a large (120 m high, with a 510 m long crest) earthfill dam. This dam, located near (2 km) the town of Yamula and near (40 km) the Kayseri province in central Turkey (approximately 320 km SE of the capital city Ankara) was designed to secure water for about 0.7 million inhabitants. The (Turkish) Ayen Energy Joint-Stock Company constructed it between 2000 and 2005. It was put into service in 2005 in order (1) to store water for the generation of electricity (storage capacity approximately 3476.00 x 10^6^ m^3^) and (2) to control river flooding. The dam was constructed from earthen material taken from the riverbed of the Kızılırmak River. The impermeable clay core of the dam is protected by semi-permeable material. The first filling period started in the December 2003 and ended in April 2005.

**Figure 1.**
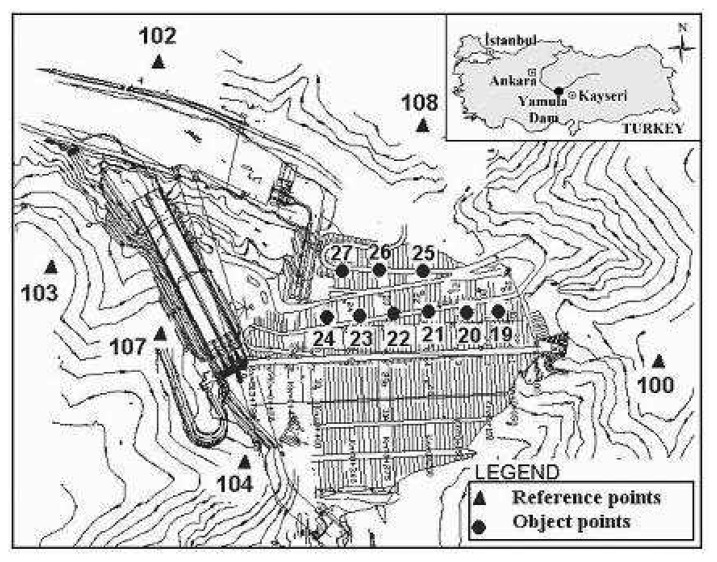
Geodetic monitoring scheme for the Yamula Dam

To ensure its structural integrity, the prevention of major damage and, especially, the safety of the public, the dam was monitored by geodetic techniques using a deformation network (Figure 1). The figure shows the distribution of reference and object points of the geodetic monitoring scheme. The aim of the geodetic deformation monitoring system of the Yamula dam is to detect possible vertical and horizontal displacements. In addition, with properly designed monitoring surveys, the second aim of the deformation monitoring system is also to determine the actual deformation mechanism and explain the causes of deformation in case of an abnormal behavior of the investigated object. The geodetic deformation monitoring system includes a number of object points on the dams and a network of local reference stations with respect to displacements of the object points are to be determined. Monitoring network consists of six reference stations (100, 102, 103, 104, 107, 108) established surroundings of the dam and of nine object points (19, 20, 21, 22, 23, 24, 25, 26, 27) on the surface of the dam's downstream face.

The original data were recorded by hand, and gross errors removed. The deformation measurements of the dam involved four measurement campaigns. The data were measured using a Total Station (Sokkia 530R). The manufacturer specifies the standard deviation of the distance measurements as ±(2 mm + 2 ppm). The deformation network was designed to detect displacements of targeted points on the downstream faces of the dams with an accuracy of 10 mm at the 95% confidence level. The deformation network was evaluated with the least squares adjustment method using both vertical (z coordinate) and horizontal (x and y coordinate) data separately. The accuracy of the displacements was calculated as ± 9 mm (maximum value) from network adjustments. The first campaign was carried out in December 2003, the second in March 2004, the third in November 2004 and the last one in April 2005. These measurements were all carried out during the first filling of the dam.

## 3. Dynamic analysis

The first filling period of a dam is the most dangerous and interesting period in a dam's life. At the reservoir filling stage two main effects must be considered: pressure of water and effect of wetting [2, 3]. In this model, as an example, the effect of the water pressure on the dam settlement during the first filling of the reservoir is shown with the geodetic monitoring results. An attempt was made to correlate the dam settlement and with water level. For this, it was assumed that the relationship between water level and the dam settlement was linear. Using this approach, a new model “ *x* = *f* (*t*,*WL*) “was developed. Here *WL* represents reservoir level which is one of the causes of the vertical displacements affecting the point positions on the dam and is a dynamic variable. If *x* = *f* (*t*,*WL*) is expanded with a Taylor series to the first degree,

(1) $$\text{x}\;(t_{i}\;,\;WL_{i})=\text{x}\;(t_{i-1}\;)+{\frac{\partial x}{\partial WL}|}_{\;(WL_{i-1}\;)}\Delta WL$$

(2) $$\text{x}\;(t_{i}\;,\;WL_{i})=\text{x}\;(t_{i-1}\;)+{\text{b}}_{\;(WL_{i-1}\;)}\Delta WL$$

where Δ*WL* and *t* are the difference of reservoir water levels and period of time between the two periods; and **b** is the water level parameters. The one-dimensional dynamic model consisting of position and water level can be written as below. In Equation (3), the unknown movement parameters consist of position and water level (first derivative of position according to water level changes). The two unknown parameters can be calculated using the Kalman-Filter technique with two measurement periods. In the Kalman-Filter technique, the movement parameters at the present time are predicted with those of the preceding (*t*_i-1_) period. Finally, the filtered (adjusted) parameters are computed, combining the predicted information and the measurements at the *t*_i_ period. To compute the movement parameters of the points with the Kalman-Filter technique, equations of position and water level can be written as below.

(3) $${\text{x}}_{j}^{\;(i\;)}={\text{x}}_{j}^{\;(i-1\;)}+\;(WL_{i}-WL_{i-1}\;)\;{\text{b}}_{\text{x}j}$$

(4) $${\text{b}}_{\text{x}j}^{\;(i\;)}={\text{b}}_{\text{x}j}$$

Equations (3) and (4) can be represented in matrix form, as given in

(5) $${\bar{Y}}_{i}={[\begin{array}{c} \text{x}\\ {\text{b}}_{\text{x}} \end{array}]\;}_{i}=\left[\begin{array}{cc} I & I\;(WL_{i}-WL_{i-1}\;)\\ 0 & I \end{array}\right]\;{[\begin{array}{c} \text{x}\\ {\text{b}}_{\text{x}} \end{array}]\;}_{i-1}$$

or in a shorter form

(6) $${\bar{Y}}_{i}=T_{i\;,\;i-1}{\hat{Y}}_{i-1}$$

where *Y̅_i_* = predicted state (position, water level) vector at period *t_i_*; *Y*ˆ*_i-1_* = state vector at period *t_i-1_*; *T_i,i_*_-1_ = transition matrix and *I* = unit matrix. Equation (6) is the prediction equation, which is the basic equation of a Kalman-Filter; *w* = constant violator acceleration vector and *N* = the system noise vector. *w* cannot be measured as a rule, so it can be taken as zero. *N* is the last column of the *T* matrix between periods *t_i_* and *t_i-1_*. The prediction equation and covariance matrix in Equation (6) can be rewritten as

(7) $${\bar{Y}}_{i}=T_{i\;,\;i-1}{\hat{Y}}_{i-1}+N_{i\;,\;i-1}w_{i-1}$$

(8) $$Q_{\bar{Y}\bar{Y}\;,\;i}=T_{i\;,\;i-1}Q_{\hat{Y}\hat{Y}\;,\;i-1}T_{i\;,\;i-1}^{T}+N_{i\;,\;i-1}Q_{ww\;,\;i-1}N_{i\;,\;i-1}^{T}$$

where *Q_YˆYˆ,i-1_* =cofactor matrix of the state vector; and *Q_ww_*,*_i_*_-1_ = cofactor matrix of the system noise at time *t_i-_*_1_. *Q_ww_*,*_i_*_-1_ can be predicted as follows.

(9) $$Q_{ww\;,\;i-1}=4{\;(t_{i}-t_{i-1}\;)}^{-4}Q_{\hat{Y}\hat{Y}\;,\;i-1}$$

The adjustment of the problem can be expressed in matrix form as

(10) $$l_{i}+v_{l\;,\;i}=A_{i}{\hat{Y}}_{i}$$

where *l_i_*, *v*_1_,*_i_*, *A*, and *Yˆ_i_* = measurements in epoch *i*, residuals, coefficients matrix, and state vector at time *t_i_*, respectively. The functional and stochastic models for the Kalman-Filter technique combining Equations (7) and (10) can be written in matrix form as

(11) $$\left[\begin{array}{c} {\bar{Y}}_{i}\\ l_{i} \end{array}\right]=\left[\begin{array}{c} I\\ A_{i} \end{array}\right]{\hat{Y}}_{i}-\left[\begin{array}{c} v_{\bar{Y}\;,\;i}\\ v_{l\;,\;i} \end{array}\right];Q_{i}=\left[\begin{array}{cc} Q_{\bar{Y}\bar{Y}\;,\;i} & 0\\ 0 & Q_{ll\;,\;i} \end{array}\right]$$

The model is solved and the movement parameters and their cofactor matrix are computed. Thus, with the Kalman-Filter technique, the two unknown parameters can be computed with two measurement periods [4-11]

As mentioned above, the parameters of position and water level are included in this process. The results of a global test of the model are shown in Tables 1 and 2, where, *a priori* variance (s_0_) was computed in a preliminary network adjustment. *A posteriori* variance (m_0_) was computed from the model. ${\text{T}=\text{m}}_{0}^{2}/{\text{s}}_{0}^{2}$. q is the *F*-distribution value. According to [12], if T<q, the global test is valid. As can be seen from Tables 1 and 2, all global test values are smaller than the α-percentage point of the *F*-distribution value (q) for a confidence level of α=0.05. Thus, the model can be viewed as accurate enough for this confidence level. That is, the global tests of the developed model are valid.

Because there is not any significant displacement in the x coordinates, these are not taken into consideration in this model. The movement parameters [vertical and horizontal displacement, (only y coordinate), water level] were computed using the dynamic method in one dimension and the results of the object points for the December 2003-November 2004, and December 2003-April 2005 are given in Table 3 for vertical displacements, and Table 4 for horizontal displacements. Because no significant settlement was determined, results of the object points for the periods of December 2003-March 2004 aren't given. Here, every parameter was divided by its standard deviation, and test values (T_z_, T_bz_, T_y_, T_by_) were computed. These values were compared with the t-distribution value (q_t_) to evaluate whether they were significant or not [13]. Where parameters have significantly changed, a (+) sign is shown; otherwise, a (−) sign is shown in decision column.

## 4. Discussion

Deformation analysis results of the dynamic model for object points located on the dam are shown in Tables 3 and 4. These tables indicate that all object points except for 19, 20, and 27 on the dam showed significant movements. Results can be noted that displacements and water level effects are maximum at the middle of the dam.

The dynamic model contains a water level parameter, which shows the physical effect of the reservoir water level on the displacements of object points. The water level parameters have physical meanings. The sign of the water level parameter is significant to be able to interpret the effect of the reservoir water level on the settlements. When analyzing the sign and the magnitude of this parameter, the effect of water level on point settlements can be determined. If “the sign of the water level parameter in decision column is positive”, a rise in the reservoir level causes settlements. If “the sign of the water level parameter in decision column is negative”, there are no settlements. When examining the water level parameters, it can be seen from Table 3 and 4 that the signs of the water level parameter in decision column except for 19, 20, and 27 are positive.

**Figure 2.**
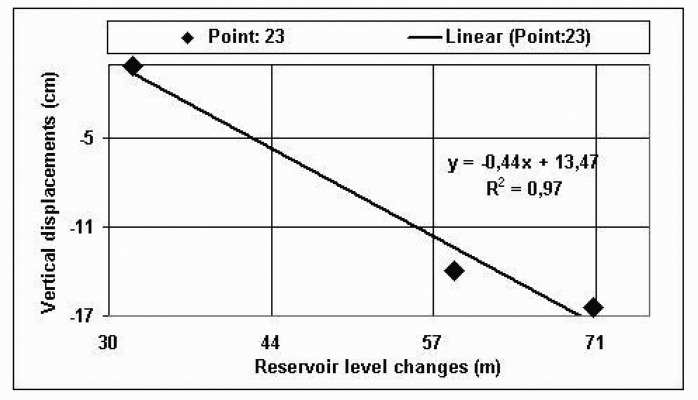
Relationship between the reservoir level and the vertical displacements at the point 23

The dynamic model shows the relationship between the rise in reservoir water level and the observed structural deformation of the dam. This relationship had been assumed as linear. An attempt was made to correlate the vertical displacements of object points and water level. In order to verify this assumption, the squares of the correlation coefficients were computed in order to find the relationship between the reservoir level and the point displacements. A graphic (Figure 2) was drawn for point 23 as an example. The graphic shows the relationship between the reservoir level and computed subsidence. The results of square of correlation coefficients for point 23 are given in Table 5. Where, WL, z, and y are the water level changes, vertical displacements and horizontal displacements between the measurement epochs, respectively. R^2^ in Figure 2 is the square of correlation coefficient. R^2^ gives the proportion of sample variety in dependent variable (displacements) that is explained by independent variable (the rise in the reservoir level). For point 23, R^2^ means that 97% of the variability in the dependent variable is explained by the independent variable and 3% is unexplained. R^2^ values for the moving points (21, 22, 23, 24, 25, and 26) are seen in Table 5 and 6. Table 6 shows results of square of correlation coefficients for moved points.

As shown in Figure 2 and Tables 5 and 6, there is an apparent linear relation between the dam settlement and the rise in the reservoir level. In addition, there was evidence of the rise in water level in the magnitude of the displacements.

**Figure 3.**
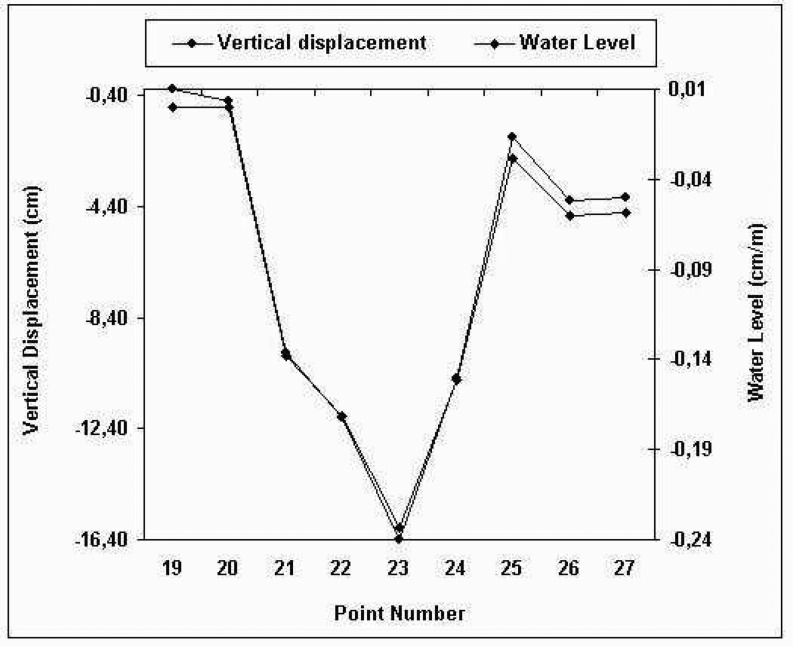
Relationship between water level parameter and the vertical displacements

This situation can be seen in Figure 3. In Figure 3, the relationship between the water level parameter and displacements was established for the period of December 2003- April 2005 (data from Table 3 for Period: December 2003- April 2005).

Relationships for all measurement periods are given in Table 7. When examining relationships between displacements and water level parameters, it can be seen (Figure 3 and Table 7) that there is a strong harmony. This means that the rising water level increases the subsidence of all object points (except for 19, 20 and 27). That is, all object points (except for 19 and 29) were affected by the rise in water level during to first filling period.

## 5. Conclusions

This article deals with the modelling of the relationships between displacements and the reservoir water levels based on a new dynamic analysis method developed for the Yamula Dam. This analysis studies and identifies how the rising reservoir level affected the dam settlement during the first filling period. The available 1.5-year four period records of the Yamula Dam indicated that all object points (except for 19, 20, and 27) were unstable. The analysis of the reservoir water level changes by the dynamic model clearly indicates that the reservoir water level changes are an important triggering factor for the Yamula Dam deformations. The developed dynamic analysis method mentioned above is capable of determining the relationships between the displacements and the rise in reservoir water. With the identified model, the simulation of the dynamic behaviour of the dam is possible considering the rise in the reservoir water. As expected, the dam was affected by the reservoir water level changes. The presented examples of modeling the dam deformation due to water pressure show that the predicted displacements are of the magnitude that can easily be detected by geodetic measurements, so, a more realistic deformation analysis can be done with the developed dynamic model which determines the causes of the deformation.

## Figures and Tables

**Table 1.** Statistical tests of the dynamic model for vertical displacements

Global Test for December 2003-March 2004		Global Test for December 2003-November 2004		Global Test for December 2003- April 2005	

s_0_	0.646	s_0_	0.646	s_0_	0.646
m_0_	0.952	m_0_	0.889	m_0_	0,658
T	2.172	T	1.894	T	1.037
q	2.596	q	2.596	q	2.596

T<q Model is valid		T<q Model is valid		T<q Model is valid	

**Table 2.** Statistical tests of the dynamic model for horizontal displacements

Global Test for December 2003-March 2004		Global Test for December 2003-November 2004		Global Test for December 2003- April 2005	

s_0_	0.626	s_0_	0.626	s_0_	0.626
m_0_	0.491	m_0_	0.988	m_0_	0.709
T	1.627	T	2.493	T	1.282
q	2.596	q	2.596	q	2.596

T<q Model is valid		T<q Model is valid		T<q Model is valid	

**Table 3.** Vertical movement parameters

^a^P.N.	Vertical Displacements (z) cm			Water Level cm / m				Vertical Displacements (z) cm			Water Level cm / m		

	^b^**z**	**T**_z_	Dec.	^c^**b**_z_	**T**_bz_	Dec.		^b^**z**	**T**_z_	Dec.	^c^**b**_z_	**T**_bz_	Dec.

19	-0.63	0.64	-	0.00	0.03	-		-0.17	0.26	-	0.00	0.01	-
20	-0.88	0.92	-	0.00	0.04	-		-0.61	0.95	-	0.00	0.04	-
21	-11.27	4.26	+	-0.20	4.08	+		-9.65	5.46	+	-0.14	5.14	+
22	-12.20	4.45	+	-0.21	4.28	+		-11.98	6.55	+	-0.17	6.19	+
23	-13.98	4.87	+	-0.24	4.71	+		-16.38	8.55	+	-0.23	8.14	+
24	-7.83	2.66	+	-0.14	2.61	+		-10.59	5.38	+	-0.15	5.15	+
25	-0.14	0.16	-	0.00	0.01	-		-1.92	1.17	-	-0.03	1.12	-
26	-0.37	0.40	-	0.00	0.02	-		-4.19	2.48	+	-0.06	2.35	-
27	-0.29	0.30	-	0.00	0.01	-		-4.06	2.38	-	-0.06	2.25	-

**Period: December 2003-November 2004**								**Period: December 2003- April 2005**					

q_t_=2.45 T> q_t_(+) T< q_t_ (-)													

a Point number,

b Vertical displacements,

c Water level parameter (cm/meter),

Dec.: Decision

**Table 4.** Horizontal movement parameters

^a^P.N.	Horizontal Displacements (y) cm			Water Level cm / m				Vertical Displacements (z) cm			Water Level cm / m		
	^b^**y**	**T**_y_	Dec.	^c^**b**_y_	**T_by_**	Dec.		^b^**y**	**T**_y_	Dec.	^c^**b**_y_	**T**_by_	Dec.
19	0.16	0.22	-	0.00	0.02	-		-0.04	0.08	-	0.00	0.01	-
20	-0.70	1.05	-	0.00	0.09	-		-0.81	1.68	-	0.00	0.15	-
21	-6.79	4.70	+	-0.22	4.35	+		-7.63	7.36	+	-0.24	6.72	+
22	-9.47	6.70	+	-0.30	6.16	+		-10.44	10.30	+	-0.33	9.40	+
23	-10.47	7.38	+	-0.33	6.67	+		-11.48	11.29	+	-0.36	10.13	+
24	-7.13	4.87	+	-0.23	4.33	+		-8.67	8.27	+	-0.27	7.22	+
25	-0.48	0.80	-	0.00	0.07	-		-2.49	2.96	+	-0.08	2.62	+
26	-0.64	1.20	-	0.00	0.11	-		-4.33	5.21	+	-0.14	4.69	+
27	0.03	0.05	-	0.00	0.00	-		-0.72	0.86	-	-0.02	0.82	-
**Epoch: December 2003-November 2004**								**Epoch: December 2003- April 2005**					
q_t_=2.45 T> q_t_(+) T< q_t_ (-)													

a Point number,

b Horizontal displacements,

c Water level parameter (cm/meter),

Dec.: Decision

**Table 5.** Displacements and reservoir water levels for point 23.

**Measurement periods**	WL (m)	z (cm)	y (cm)

December 2003-March 2004	31.99	-0.08	-0.13
December 2003-November 2004	58.72	-10.47	-13.98
December 2003-April 2005	70.33	-11.48	-16.38

Square of correlation coefficient (R2)		0.97	0.95

**Table 6.** The square of the correlation coefficients for the moving points

**Point Number**	**square of correlation coefficient**	

	**z**	**y**

21	0.82	0.96
22	0.90	0.95
23	0.97	0.95
24	1.00	0.98
25	0.65	0.71
26	0.63	0.96

**Average**	**0.83**	**0.92**

**Table 7.** The square of the correlation coefficients for different periods

**Measurement epochs**	**Square of correlation coefficient (R^2^)**	

	**z**	**y**

December 2003-March 2004	0.99	0.98
December 2003-November 2004	0.99	0.99
December 2003-April 2005	0.99	0.99
